# A Delphi consensus among experts on assessment and treatment of disruptive mood dysregulation disorder

**DOI:** 10.3389/fpsyt.2023.1166228

**Published:** 2024-01-08

**Authors:** Assia Boudjerida, Jean-Marc Guilé, Jean-Jacques Breton, Xavier Benarous, David Cohen, Réal Labelle

**Affiliations:** ^1^Department of Psychology and Centre for Research and Intervention on Suicide, Ethical Issues and End-of-Life Practices, Université du Québec à Montréal, Montréal, QC, Canada; ^2^Department Head, Child and Adolescent Psychiatry, EPSM Somme and CHU Amiens, Picardie Jules Verne University, Amiens, France; ^3^Department of Psychiatry, Rivière-des-Prairies Mental Health Hospital, Université de Montréal, Montréal, QC, Canada; ^4^Department of Child and Adolescent Psychopathology CHU Amiens-Picardie, Amiens, France; ^5^Department Head, Child and Adolescent Psychiatry, Public Assistance-Hospitals of Paris, APHP, Pitié-Salpêtrière Hospital Group and National Center for Scientific Research-Joint Research Unit, Institute for Intelligent and Robotic Systems Sorbonne Université, Paris, France

**Keywords:** disruptive mood dysregulation disorder, Delphi study, experts consensus, mental health, quality of care

## Abstract

**Objective:**

The aim of this study was to explore consensus among clinicians and researchers on how to assess and treat Disruptive Mood Dysregulation Disorder (DMDD).

**Methods:**

The Delphi method was used to organize data collected from an initial sample of 23 child psychiatrists and psychologists. Three rounds of closed/open questions were needed to achieve the objective.

**Results:**

Fifteen experts in the field completed the whole study. Finally, 122 proposals were validated and 5 were rejected. Globally, consensus was more easily reached on items regarding assessment than on those regarding treatment. Specifically, experts agreed that intensity, frequency, and impact of DMDD symptoms needed to be measured across settings, including with parents, siblings, peers, and teachers. While a low level of consensus emerged regarding optimal pharmacological treatment, the use of psychoeducation, behavior-focused therapies (e.g., dialectical behavior therapy, chain analysis, exposure, relaxation), and systemic approaches (parent management training, family therapy, parent–child interaction therapy) met with a high degree of consensus.

**Conclusion:**

This study presents recommendations that reached a certain degree of consensus among researchers and clinicians regarding the assessment and treatment of youths with DMDD. These findings may be useful to clinicians working with this population and to researchers since they also highlight non-consensual areas that need to be further investigated.

## Introduction

Disruptive Mood Dysregulation Disorder (DMDD) was coined as a separate diagnostic entity in the fifth edition of the Diagnostic and Statistical Manual of Mental Disorders (DSM-5). The disorder’s main criterion is persistently irritable mood punctuated by recurrent, intense temper outbursts and the diagnosis should be made for the first time between ages 6 and 18 years (see Table 1 for a summary of the DSM-5 criteria). The disorder’s prevalence in the general population has been estimated at 2.5% (1–4). This diagnosis came into being in the wake of the controversy concerning the surge in pediatric bipolar disorder (PBD) diagnoses in the United States.

**Table 1:** Diagnostic criteria for DMDD.

Severe recurrent temper outbursts manifested verbally and/or behaviorally that are grossly out of proportion in intensity or duration to the situation or provocation. The temper outbursts are inconsistent with developmental level. The temper outbursts occur, on average, three or more times per week. The mood between temper outbursts is persistently irritable or angry most of the day, nearly every day and is observable by others. Criteria A–D have been present for 12 or more months without a period lasting 3 or more consecutive months without all of the symptoms presented above. Criteria A and D are present in at least two of three settings (at home, at school, with peers) and are severe in at least one of these. The diagnosis should not be made for the first time before age 6 years or after age 18 years. By history or observation, the age at onset of Criteria A-E is before 10 years. Exclusion: episode of mania or hypomania. Symptoms do not occur exclusively during an episode of major depressive disorder and are not better explained by another mental disorder. Symptoms not better explained otherwise.

This diagnosis cannot coexist with oppositional defiant disorder, intermittent explosive disorder or bipolar disorder. It can coexist with other diagnoses, including major depressive disorder, attention-deficit/hyperactivity disorder, conduct disorder and substance use disorders.

PBD was first proposed in 1995 by researchers who were keen to identify this disorder early and who postulated that mania might occur in early childhood but differently from adulthood. The research hypothesis presented two phenotypes: multiple mood episodes per day (5) or chronic irritability generally without elevated mood (6). Whereupon, according to Moreno et al. (7), the number of medical visits associated with bipolar disorder diagnoses in children had increased fortyfold from 1994 to 2003. Some authors indicate that “the increase in rates of bipolar disorder in children has been controversial and it remains unclear whether this trend represented an increase in recognition, an increase in incidence, or a broadening of the application of the diagnostic criteria” (8). Yet, the PBD diagnostic increase remains mostly confined to the United States (9). Some explanations can be given. First, it seems that in the United States, diagnoses of more serious conditions allow for greater reimbursement and access to resources than less serious ones (9). Therefore, if in doubt, it seems more useful to diagnose a PBD than an oppositional defiant disorder (ODD) for example. Second, the PBD increase could also reflect the problem of reification. It’s a cognitive bias which consists in considering an abstract idea as a concrete entity. Some researchers evoke that the model of the DSM developed to foster interrater reliability facilitates reification, which leads to a false sense of validity (10).

Nevertheless, in 2003, Leibenluft and colleagues at the National Institute of Mental Health (NIMH) proposed a series of studies to document the links between chronic irritability and bipolar disorder (11). Research on this controversy was largely motivated by its treatment implications (12). These researchers, named the syndrome “Severe Mood Dysregulation” (SMD) to identify children with chronic irritability associated with temper outbursts and non-episodic symptoms. In a systematic review, this team concluded that chronic irritability in childhood was not a symptom associated with bipolar disorder but rather unipolar disorder (13). Which should lead to a considerable paradigm shift for treatments. Thus the DSM-5 Task Force (1) deemed the NIMH studies to be sufficiently conclusive to include SMD as a new diagnostic entity among mood disorder under a new name: Disruptive Mood Dysregulation Disorder (14). DMDD was placed in the depressive disorders section based on a series of arguments that underscored its continuity with depression in adulthood in terms of family history, neurocognitive disturbances, genetic factors and, particularly, on outcome studies (13, 15).

However, DMDD’s validity as an independent diagnostic entity has been questioned for different reasons. First, the extrapolation of data on SMD to DMDD is not necessarily obvious. A study showed that only 58% of young people with SMD also met the criteria for DMDD, and only 47% of those with DMDD met the criteria for SMD (4). Second, a study on DSM-5 field trials indicated that DMDD has low level of evidence on the temporal stability of symptoms and the reliability of diagnostic criteria (16). Third, the very high level of comorbidity of DMDD with other psychiatric disorders (varies between 60 and 95% according to certain studies) (17) is an important limit to the validity of the diagnosis. In fact, the main criteria of DMDD (outbursts and irritability) are also found in other disorders (ODD, attention-deficit/hyperactivity disorder, anxiety, depression) which complicates things. Hence, some authors suggest that “This lack of phenomenological distinction means that the diagnosis has failed to achieve its primary goal, namely, to inform treatment” (18). In fact, alternatively to DMDD’s diagnosis, in ICD-11, chronic irritability accompanied by outbursts, is more of a specifier in ODD (19). Additionally, outbursts are core symptoms in intermittent explosive disorder (IED) in both DSM-5 and ICD-11. Thus, several experts in the field of irritability indicate that “The lack of a standard definition or diagnostic home has relegated outbursts to nosological orphanhood” (20). It is therefore a fairly recent and still evolving field of study.

Even so, in clinical practice, youths who fit the DMDD profile have recurrent temper outbursts that have a real impact on the quality of their family and peer relationships, and on their academic performance (1). They often present comorbidities, particularly neurodevelopmental disabilities (e.g., Attention deficit with or without hyperactivity-ADHD) (21, 22). Children with DMDD experience a higher level of social problems and are more often suspended from school than other students are (3). Moreover, compared with youths with other psychopathologies, they are more likely to report impairment in activities of daily life and more suicidal ideation and behaviors (2). Finally, youths with DMDD make greater use of health services (23). The addition of this diagnosis to the DSM-5 (1) and the knowledge about the individual and collective burden associated with this disorder have led us to explore how clinicians and researchers perceive, assess and treat these youths. Ten years after the publication of the latest DSM, there is still no consensus in this regard (24).

As controversies remain on the diagnostic and therapeutic approaches that should be prioritized in the care of DMDD patients, the current study focusses on expert’ opinions. Our goal was to combine the experiences of these professionals to offer consensual recommendations on DMDD.

## Materials and methods

### The Delphi method

The Delphi method serves to collect the opinions of experts on a specific subject. It has been used to build a consensus among experts on numerous clinical protocols and has proved effective in the field of mental health (25). The idea is to query a group of experts by way of self-administered questionnaires, in an iterative and interactive manner, without any direct communication between them (26–28). Moreover, it is a simple, cost-effective, flexible method that eliminates geographical boundaries, enables knowledge sharing, and allows freedom of expression through the use of anonymity (29). Under the classic version of the method, experts are queried four times (30), first through open-ended questions on a given issue and then through closed-ended questions (31). However, to minimize time burden and participant attrition, many authors have proposed cutting the rounds of questioning to two or three (32–34). For the purposes of our study, we opted for three rounds.

The scientific literature does not propose any guidelines regarding the number of participants that should be involved (25). This depends on several factors, including the characteristics of the subject under investigation and the number of experts potentially available (35). Many exponents of the Delphi method, including Parenté and Anderson-Parenté (36) and Linstone and Turoff (27), have deemed a minimum of 10 participants to be enough when the sample is a rather homogenous group of experts. Other authors have suggested involving from 8 to 12 participants for a panel of specialized experts (37, 38). In our study, all of the participants recruited were either psychiatrists or psychologists and experts in the field. They constituted a fairly uniform group.

### Definition of consensus

To date, there are no clear guidelines regarding the definition of consensus (35). Some researchers have deemed a consensus reached with 51% of respondents in agreement whereas others have placed the bar at 70% or 80% (39). According to Sumsion (40) and McKenna, Hasson (41), for example, 70% of respondents in agreement would constitute a sufficiently strong consensus. The literature recommends, also, using a Likert scale and measures of central tendency, such as the median, to give participants feedback, regardless of how consensus is defined (42–44). These elements were taken into consideration in conducting our study.

### Procedure

First, few months before beginning the study, a pilot run was carried out to test the use of the online platform and fine-tuning the wording of questions in English and French as previously recommended (35, 39, 45). For the first round, in order to reproduce the concept of brainstorming, as recommended in the Delphi Method (46), four open-ended questions were formulated as follows: (i) Name at least three key elements that you consider essential for an effective assessment of DMDD; (ii) Name at least three key elements of pharmacological intervention for DMDD; (iii) Name at least three key elements of psychosocial intervention for DMDD; (iv) Name at least three main targets of intervention for DMDD. A dozen student-researchers completed the first two rounds of the survey and evaluated the questionnaires in terms of design and clarity. Minor changes were made to the questionnaires thanks to this feedback. For example, comment boxes were added at various points to catch participants’ reactions and opinions. According to Tremblay-Boudreault and Dionne (47), such adaptations tends to enhance the overall level of experts’ participation.

Next, three-round survey was carried out online from July 2021 to May 2022. In the first round, participants received a questionnaire with three parts: (i) consent form; (ii) list of sociodemographic information; and (iii) four open-ended questions on assessment and intervention practices. Thus, the experts could identify what, in their opinion, were the key elements in the assessment and treatment of DMDD. In the second round, participants received a list of items proposing different practices based on the responses collected in the first round. They were asked to rate how much they agreed with these proposals. Items where a consensus (in agreement or disagreement) existed were then either retained or rejected. In the third round, participants were asked to re-assess how much they agreed with items on which there was no consensus after being informed of where the group stood (median) on these following the second round.

### Participants

Participants had to meet three criteria: (i) to be a psychiatrist or psychologist; (ii) to have had an article published in a scientific journal about DMDD or to self-identify as treating youths with main symptoms of DMDD (criterion A, C, and D; see Table 1 for a summary of the criteria); and (iii) to be fluent in English or French. Potential participants were identified through existing publications on the subject of DMDD and snowball sampling. In other words, participants selected on the basis of publications were asked to recommend colleagues who also worked with this client group (48). In the end, 103 experts were solicited by email.

### Ethical considerations

In the first round of the study, prior to any question, participants had to confirm having read the consent form, indicate that they met the inclusion criteria, and declare no conflict of interest. In this regard, each expert was informed in writing that he could not receive a benefit (financial, moral or professional) from a third party (pharmaceutical company or health company) in the choice of the selected proposals. In fact, the only contribution, but still valuable, was to advance knowledge. Then, participants could choose whether to be named and thanked in articles derived from the study. This was meant as a token of appreciation for their time spent on the project. The experts were free to withdraw from the study at any time without justification. The project was approved by the Human Research Ethics Board of Université du Québec à Montréal, in Canada (Approval no. 4953).

### Descriptive statistics

**Figure 1:**
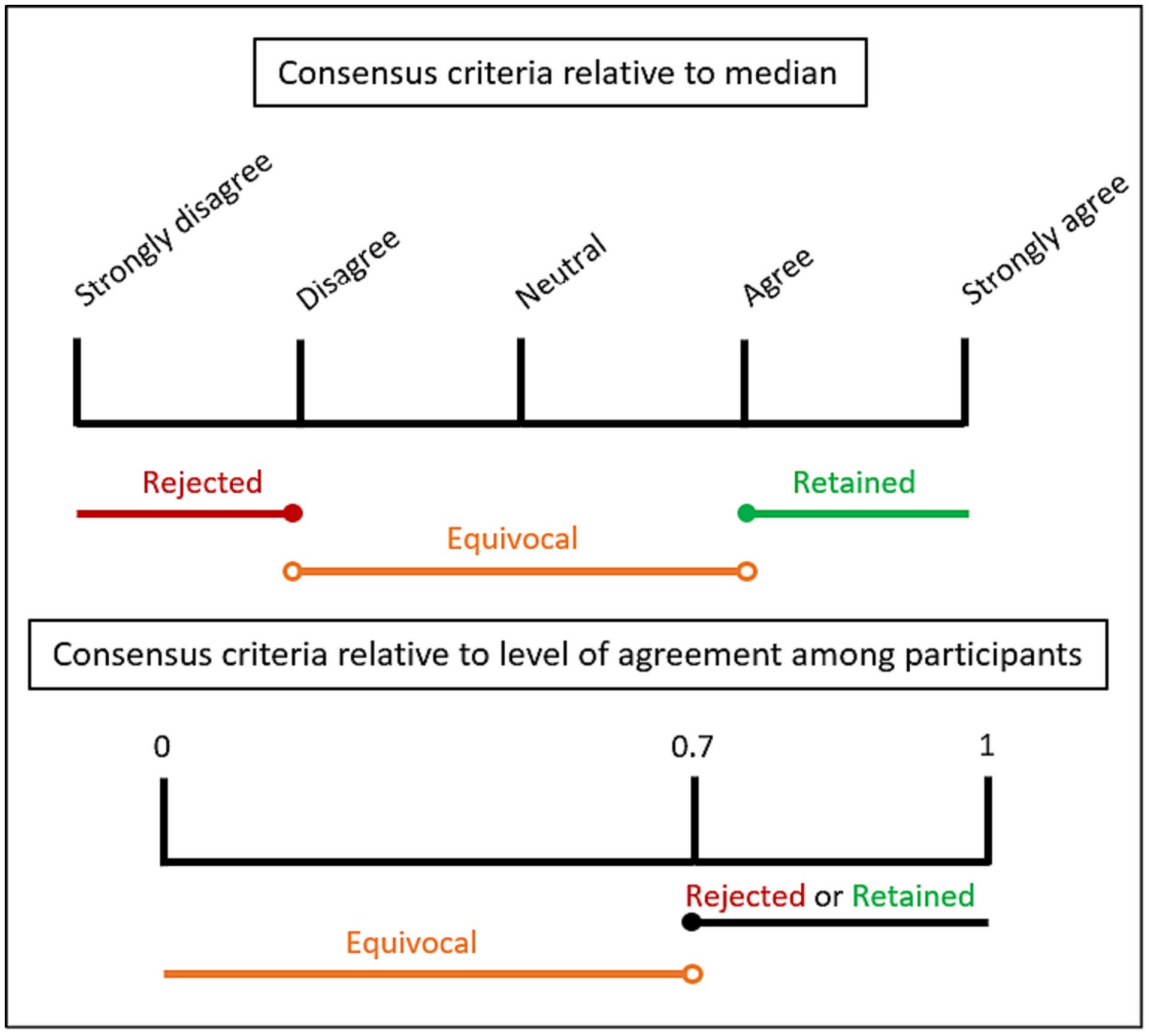
Consensus level criteria.

The questionnaires were hosted on LimeSurvey, a free and open source online statistical survey web app, and statistical analyses were performed using Microsoft Excel. The open-ended responses in the first round were subjected to conventional content analysis (49, 50). Accordingly, they were parsed and grouped under themes that emerged during data analysis. An independent researcher with no connection to this study repeated the data analysis process for the purpose of assessing inter-judge agreement (51). This allowed us to create the short proposals that were rated in the second round. Participants had to indicate how much they agreed with each item on a five-point Likert scale, though they could abstain from responding if they wanted. Statistical analyses in the second round consisted in assessing level of agreement among participants. For the purposes of our study, items were retained by consensus when at least 70% of the participants responded *agree* or *strongly agree* (41). Items were rejected by consensus when at least 70% of the participants responded *disagree* or *strongly disagree.* Otherwise, items were considered equivocal and were re-assessed in the third round. Items could be considered equivocal also if the group median fell close to the *neutral* position on the Likert scale. Figure 1 illustrates the criteria used to determine consensus. These analyses were repeated after the third round.

## Results

### 1st round

**Figure 2:**
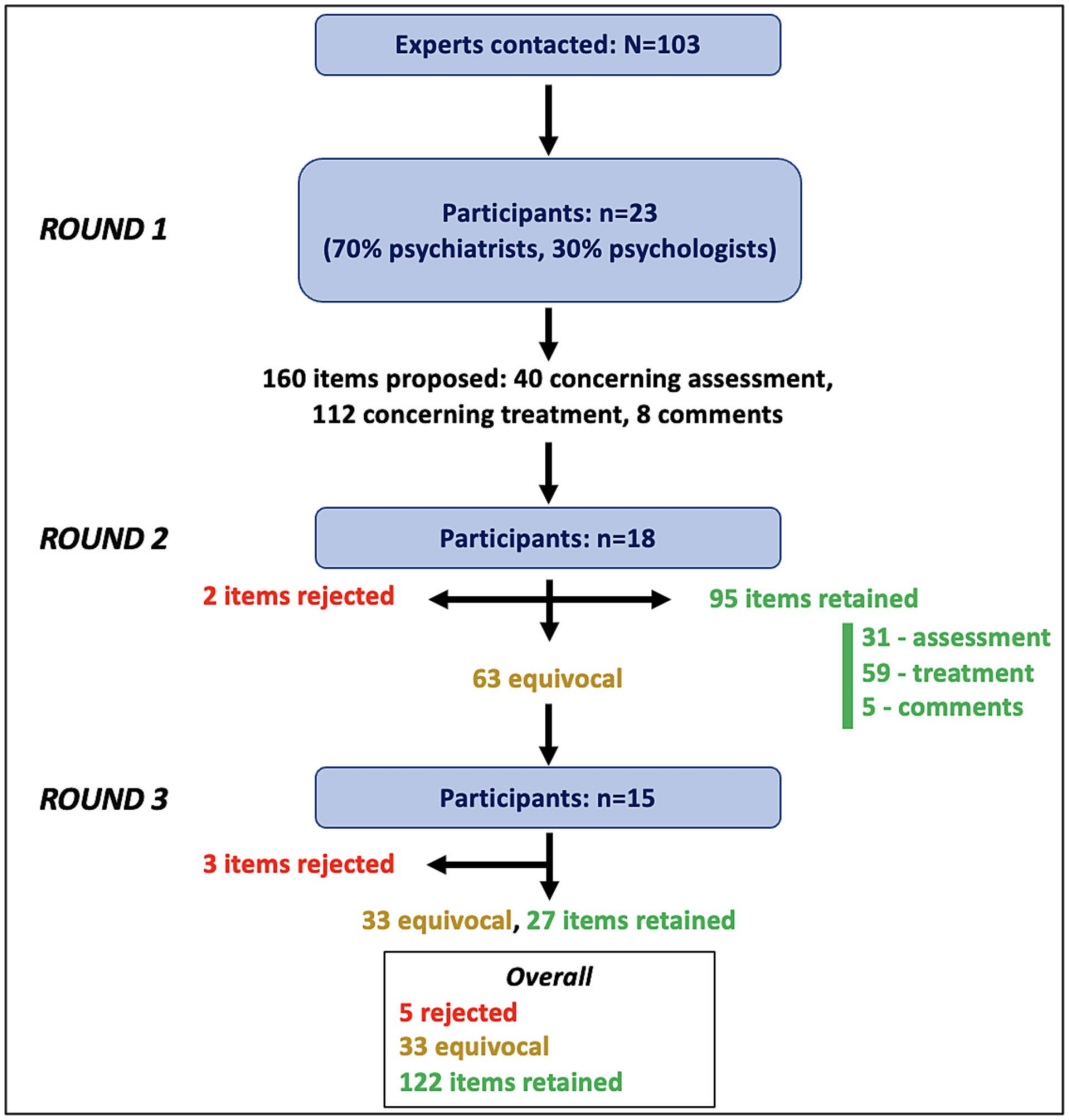
Flowchart illustrating three rounds of survey.

Of the 103 experts contacted, 23 completed the first round of the study and, of these, 43% were women. The majority of the respondents were psychiatrists (70%) and the rest were fully qualified psychologists. Furthermore, 70% of the experts were both researchers and clinicians, 17% did research only, and 13% were clinical practitioners only. Moreover, 87% worked in the public sector, part of which have a dual private-public practice. Regarding years of practice, 13% of respondents reported less than 5 years, 43% reported 5–14 years, 22% reported 15–24 years, and 22% reported 25 years or more. Finally, participants came from eight different countries: United States (*n* = 8), Canada (*n* = 4), France (*n* = 4), Australia (*n* = 2), Turkey (*n* = 2), Switzerland (*n* = 1), Ireland (*n* = 1), and Brazil (*n* = 1). Figure 2 is a flowchart showing participant retention and number of proposals retained after each round. The responses provided by the experts allowed us to formulate 160 proposals regarding DMDD. Of these, 40 related to assessment, 112 to treatment (26 to intervention targets, 40 to psychosocial interventions, and 46 to pharmacological interventions), and 8 to general comments.

### 2nd round

Eighteen of the 23 experts completed the second round, for a retention rate of 78%. Of these, 39% were women, 61% were psychiatrists, 72% of the experts were both researchers and clinicians, 22% did research only, and 6% were clinical practitioners only and 89% worked in the public sector. Regarding years of practice, 11% of respondents reported less than 5 years, 50% reported 5–14 years, 17% reported 15–24 years, and 22% reported 25 years or more. Finally, there are still participants from the 8 countries named above. Of 160 proposals submitted, 95 were retained and 2 were rejected by consensus. The other 63 items were considered equivocal and were submitted again in the third round. More specifically, the vast majority of the assessment items were retained (31/40). Where treatment was concerned, aside from the item regarding sensorimotor integration, the proposals regarding intervention targets were all retained (25/26). As for psychosocial interventions, responses were rather mixed: half of the items were retained and the other half were equivocal (20/40). Regarding pharmacological interventions, almost no item was retained other than general concepts (14/46). In this section, two items were rejected outright: use of oxcarbazepine and use of lithium. Finally, a few comments were retained following this round (5/8).

### 3rd and final round

Fifteen experts completed the final round of the study, which results in a retention rate of 83% relative to the second round and of 65% relative to the first. Of these, 40% were women, 60% were psychiatrists, 80% of the experts were both researchers and clinicians, 20% did research only, none were clinical practitioners only, and 87% worked in the public sector. Regarding years of practice, 7% of respondents reported less than 5 years, 53% reported 5–14 years, 13% reported 15–24 years, and 27% reported 25 years or more. At this point, there were no more experts from Brazil, so the participants came from seven different countries: United States (*n* = 5), Canada (*n* = 2), France (*n* = 3), Australia (*n* = 1), Turkey (*n* = 2), Switzerland (*n* = 1), and Ireland (*n* = 1). Of the 63 items proposed again in this round, 27 were retained by consensus, 33 remained equivocal, and 3 were rejected by consensus.

After the third round (Figure 2), of the 40 assessment proposals, 36 were retained. Moreover, as in the second round, of the 26 DMDD intervention targets, all were retained except for the one regarding sensorimotor integration. Of the 40 psychosocial intervention items, 32 were retained and the rest remained equivocal. Of the 46 pharmacological intervention proposals, 22 were retained and 5 were rejected. Finally, 7 of 8 comments were validated by consensus.

In sum, consensus was more easily achieved on items concerning assessment than on those concerning treatment. Specifically, the experts approved the idea that the intensity, frequency and consequences of DMDD had to be measured across different contexts (e.g., with parents, siblings, peers, teachers). The pharmacological proposals were the most controversial whereas the items regarding psychoeducation and behavioral interventions were more consensual (e.g., dialectical behavior therapy, relaxation), as were those regarding family approaches (parent management training, family therapy, therapy focused on parent–child interaction). The 122 proposals retained and the 5 rejected by the group of experts are given in Table 2, along with their respective degree of agreement. The comments, made in the free text boxes, retained by the participants are presented in Table 3 and the equivocal proposals are detailed in Table 4.

**Table 2:** Level of agreement on retained and rejected initial proposals.

**Assessment**		
	**Level of agreement**	
**General elements**	**Retained**	**Rejected**
Requires clinician expertise and excellent knowledge of DSM-5 criteria for DMDD	100%	
Establish clustering, severity, frequency, and developmental course of mood and temper outbursts	100%	
Assess all DSM-5 criteria and specifications and time course including age of onset	89%	
Obtain a clear description of symptoms and their duration	100%	
Obtain clinical history and chronological history of mood symptoms	100%	
Establish family history	72%	
Identify impact of symptoms in multiple settings and relationships (including with parents, siblings, peers, and teachers)	100%	
Assess impact on functioning and level of impairment	100%	
Explore accommodations in place that may affect irritability and outbursts	89%	
Identify risk and protective factors	88%	
Observe at school, at home, and during peer activities	73%	
**Components to assess in relation to temper outbursts**		
Make a clear behavioral description of severe outbursts: frequency (weekly or monthly), intensity, duration, triggers, environmental context, and pervasiveness	100%	
Target triggers (i.e., physical cues like hunger or fatigue versus other cues such as difficulty transitioning between activities)	89%	
Measure aggression	94%	
Measure impulsivity	78%	
Measure frustration intolerance	100%	
**Components to assess relative to irritability and mood**		
Complete a longitudinal assessment of baseline or typical mood state (including irritability, sadness and grouchy mood)	100%	
Define and quantify amount of time child/adolescent is irritable per week/month and in what settings, and severity of irritability (mild, moderate, severe)	94%	
Measure mood between outbursts	100%	
Measure emotional instability	89%	
**Differential diagnosis and other elements to consider**		
Screen for explanatory causes, such as rule out all conditions listed in DSM (e.g., mania, bipolar disorder, physical and mental disorders, and known contributory factors such as maltreatment, family issues, substance use, medication, and stressors)	100%	
Identify depression symptoms	100%	
Identify suicidal behavior	100%	
Identify sleep problems	94%	
Identify comorbid anxiety	94%	
Verify comorbid ADHD	100%	
Verify other comorbid neurodevelopmental or learning disorders	94%	
**Assessment tools**		
Use comprehensive clinical interview and validated measures	100%	
Use Affective Reactivity Index (ARI) for irritability	93%	
Use Children’s Depression Rating Scale (CDRS) for depression symptoms	86%	
Use Conners for ADHD symptoms	80%	
Use Child Behavior Checklist (CBCL) for emotional and behavioral problems	80%	
**People to involve in assessment**		
Establish multi-informant assessment. It is critical to get both parent and child report of symptoms, and teacher report when possible.	94%	
Include both parents (if available) to talk about how DMDD symptoms manifest and if they are more evident with one parent or the other	89%	
Consult school report cards and school records	89%	
Follow four-stage procedure for child psychiatric assessment: (a) interview parents and child together, (b) meet with parents only (have a separate professional do this, if possible), (c) conduct individual examination of child, and (d) meet with parents and child, along with all professionals involved, to sum up situation.	78%	

**Treatment**		
	**Level of agreement**	
**Intervention targets – General concepts**	**Retained**	**Rejected**
Recognize disorder as involuntary, not directed at adults (parents, teachers)	88%	
Target irritability	100%	
Target self-esteem	88%	
Reduce number of emergency room visits and hospital stays	100%	
Improve global functioning	100%	
**Intervention targets – Emotion regulation**		
Teach emotion regulation strategies	100%	
Tackle emotional lability	94%	
Improve daily mood	88%	
Improve distress tolerance	94%	
Improve frustration tolerance	94%	
Improve stress management	82%	
Reduce hostile interpretation bias	71%	
Reduce risk of suicide or self-harm	94%	
Decrease aggressive outbursts	100%	
Decrease impulsiveness	88%	
Prevent endangering oneself and others	100%	
Improve problem-solving skills	100%	
**Intervention targets – Interpersonal relationships**		
Improve parent–child relationship by promoting positive interactions	100%	
Repair interpersonal breakups, mainly with parents	88%	
Work on family maladjustment and poor dynamics	94%	
Guide parental behaviors	100%	
Help improve peer relationships	94%	
Decrease impaired social cognitions	94%	
Recognize interpersonal triggers	94%	
Improve social relationship skills	94%	
**Psychosocial intervention – General concepts**		
Use assessment as a guide, select best treatment option to target symptoms (developmental stage, situation, and comorbidity should drive choice)	94%	
Use elements of CBT and parent management training, but more research needed to tease this out	94%	
Practice cognitive behavioral therapy	94%	
Use behavioral approach	88%	
Foster a systemic approach	86%	
Foster family therapy	86%	
Foster social care with educational assistance if necessary and parenting support (intervention by social worker)	81%	
Foster intervention focused on child and parent distress and coping strategies to deal with chronic irritability	94%	
Aim for at least 8 weeks for a primary care presentation, more sessions required for more entrenched difficulties	92%	
**Psychosocial intervention - Joint interventions (child/adolescent, parents and school)**		
Involve child/adolescent, parents and sometimes even extended family and teachers	94%	
Offer psychoeducation and psychotherapy with family	93%	
Offer family intervention including learning validation	71%	
Teach chain analysis (antecedents, behaviors, consequences) to parents and child	87%	
Use Parent Child Interaction Therapy	100%	
Liaise with school community	100%	
**Psychosocial intervention - With child/adolescent**		
Use Cognitive Behavioral Therapy for child or adolescent individually	80%	
Use Cognitive Behavioral Therapy, delivered preferably in a group format (6–8 participants) for up to 12 sessions	86%	
Use Dialectical Behavior Therapy (DBT) adapted for children or adolescents (DBT-C or DBT-A)	86%	
Adapt DBT: whatever is reasonably available in individual and group format, ideally both	92%	
Use exposure-based techniques that focus on heightened reactivity to frustrative non-reward and aberrant approaches to threats	71%	
Use interventions that include teaching how to recognize somatic signals of different emotions, including anger, gauging intensity of emotions (i.e., feelings thermometer)	87%	
Teach emotion regulation and anger management strategies to child/adolescent	94%	
Foster interventions using social learning and behavioral principles	100%	
Teach coping skills	86%	
Teach relaxation techniques	86%	
Use executive functioning treatment if ADHD is comorbid	86%	
Encourage regular participation in sports or art activities as this may help children/adolescents manage emotional intensity and improve self-esteem	100%	
**Psychosocial intervention – For parents and family**		
Foster parent psychoeducation	100%	
Foster Parent Management Training (PMT)	94%	
Use parent modeling for oppositional defiant disorder, adapted somewhat to DMDD	93%	
Foster positive parenting	86%	
Focus on parent–child relationship in hope of identifying negative or coercive parenting practices	93%	
**Pharmacological intervention – General concepts**		
There is no consensus, not enough scientific evidence	73%	
It is preferable to turn to a psychiatrist specializing in mood disorders in children	80%	
Psychiatrist has the duty to know well and to inform himself about the most promising molecules in the treatment of this disorder	87%	
Target symptoms to be alleviated	93%	
Use comorbidity as guidepost to select best pharmacological agent	93%	
Monitor closely for adverse events that may exacerbate emotion dysregulation in DMDD	100%	
**Pharmacological intervention – Where to start**		
Treat comorbidities first with appropriate meds	93%	
Start with stimulant optimization if comorbid ADHD. If DMDD still present thereafter, add selective serotonin reuptake inhibitors (SSRI)	83%	
Start with SSRI if no comorbid ADHD	90%	
**Pharmacological intervention – Medication usually offered for ADHD**		
Consider stimulants if child/adolescent meets criteria for ADHD	85%	
Consider methylphenidate	77%	
Consider doses: stimulants 0.5–1.2 mg/kg/day	80%	
**Pharmacological intervention – Antidepressants (SSRI)**		
Consider anti-depressants that might lower irritable mood	92%	
Review hypothesis of efficacy of SSRI given that DMDD falls under category of depressive disorders	83%	
Consider SSRI same as when treating depression in adults if clinically significant depression	100%	
Consider fluoxetine in case of comorbid depression or anxiety symptoms	70%	
**Pharmacological intervention – Atypical antipsychotics**		
Consider antipsychotics as last resort, after other modalities, including psychotherapeutic approaches, have been tried	83%	
Consider atypical antipsychotic if marked aggression, aggressive behavior at school and family disorganization with parental exhaustion	82%	
Consider risperidone (e.g., Risperidal)	73%	
Consider risperidone in context of autism spectrum disorder	78%	
Consider aripiprazole (e.g., Abilify)	70%	
**Pharmacological intervention – Other medications**		
Consider oxcarbazepine (Trileptal)		75%
Consider lithium (blood level same as for mania)		80%
Consider cyamemazine (Tercian)		89%
Consider levomepromazine (Nozinan)		89%
Consider sodium valproate (10 mg/kg body weight)		90%
Consider symptomatic treatment of difficulty falling asleep	90%	

Percentages were rounded out.

**Table 3:** Other comments made in first round.

**Other Comments made in first round**		
	**Retained**	**Rejected**
DMDD is still controversial	80%	
Most DMDD diagnoses do not abide by DSM rules for differential diagnosis	86%	
ICD-11 construct of ODD with emotion dysregulation should be reviewed in connection with DMDD	87%	
Role of childhood trauma, attachment difficulties, and parents with mental illness, including borderline personality disorder and ADHD, are associated with DMDD and should be explored	88%	
Complex post-traumatic stress disorder and DMDD and transition of DMDD with borderline personality disorder should be explored	94%	
Issue of over-diagnosis of pre-pubertal bipolar disorder that led to DMDD construct needs to be reviewed	87%	
Participants’ views on comorbidities (especially ODD/IED) accompanying DMDD should be explored	80%	
DMDD is still controversial	80%	
Most DMDD diagnoses do not abide by DSM rules for differential diagnosis	86%	
ICD-11 construct of ODD with emotion dysregulation should be reviewed in connection with DMDD	87%	
Role of childhood trauma, attachment difficulties, and parents with mental illness, including borderline personality disorder and ADHD, are associated with DMDD and should be explored	88%	
Complex post-traumatic stress disorder and DMDD and transition of DMDD with borderline personality disorder should be explored	94%	
Issue of over-diagnosis of pre-pubertal bipolar disorder that led to DMDD construct needs to be reviewed	87%	
Participants’ views on comorbidities (especially ODD/IED) accompanying DMDD should be explored	80%	

Percentages were rounded out.

**Table 4:** List of equivocal proposals and other comments made in first round.

**Assessment**
**Differential diagnosis and other elements to assess**
Consider sensorimotor integration (Dunn’s sensory profile)
**Assessment tools**
Use K-SADS-PL DMDD Module for DMDD criteria
Use Retrospective-Modified Overt Aggression Scale (R-MOAS) for impulsive aggression
Use tests for intelligence and for learning problems (e.g., WISC-IV)
**Treatment**
**Intervention targets – General concepts**
Consider sensorimotor integration
**Psychosocial intervention - General concepts**
Use mentalization-based therapy
Use combination of individual and group approach
Aim for duration of at least six months for primary care
**Psychosocial intervention - With child/adolescent**
Foster interpersonal psychotherapy: emotion identification and regulation skills through increasing awareness of emotions. Learning to take breaks when irritability increases to de-escalate and discuss issue when calm. Improvement in communication skills. Working on interpersonal problem-solving skills.
Use cognitive restructuring
Use assertiveness training, especially for recognition of ambiguous social situations
Use assertiveness training in groups
Use ABA with functional communication training (FCT) for young children
**Pharmacological intervention – Where to start**
Use stimulants first, then SSRI, then antipsychotics/anticonvulsants if symptoms remain after treating comorbidities
**Pharmacological intervention - Medication usually offered for ADHD**
Consider atomoxetine
Consider Strattera if there are anxiety symptoms
Consider doses: atomoxetine from 0.5 up to 1.4 mg/kg/day
**Pharmacological intervention - Antidepressants (SSRI)**
Add on citalopram if unresponsive to methylphenidate
Consider Zoloft in case of comorbid depression or anxiety symptoms
Consider escitalopram in case of comorbid depression or anxiety symptoms
Consider doses: escitalopram up to 20 mg/day
Consider doses: fluoxetine up to 20 mg/day
**Pharmacological intervention - Atypical antipsychotics**
Consider risperidone in the context of impulsivity and frustration intolerance
Consider doses: risperidone 0.5 up to 3 mg/day
Consider doses: risperidone do not exceed 1 mg/day
Consider doses: aripiprazole 2.5 up to 15 mg/day (for acute management, six months)
Consider doses: aripiprazole do not exceed 1 mg/day or 5 mg/day
Consider doses: aripiprazole do not exceed 5 mg/day
Consider quetiapine
**Pharmacological intervention - Other medications**
Consider lamotrigine (up to 200 mg)
Consider clonidine (0.2–0.4 mg/day)
Consider guanfacine (2–4 mg/day)
**Other comments made in first round**
New ICD criteria make more sense than DSM-5 criteria

## Discussion

This study presents recommendations that reached some degree of consensus among researchers and clinicians regarding the assessment and treatment of youths with DMDD. The expertise of psychiatrists and psychologists shed light on issues regarding DMDD that remain unanswered in the literature. Moreover, the equivocal proposals that emerged from our study, as well as the numerous comments formulated by the participants, are grey areas to be explored in future research (Table 4). Those findings could help improve how these youths are identified and managed.

This study is not without limitations. Above all, our group of participants was rather small and was not representative of all the mental health experts of the world. It would have been interesting to have a larger sample and to be able to examine the international differences, especially considering that the DSM-5 and the ICD offer a different understanding of symptoms. Moreover, clinicians self-identified as treating patients with core DMDD symptoms. Hence, they may vary in the amount of actual hands-on clinical experience with DMDD youth. Also, while the participation rate was adequate from one round to the next, it was difficult to recruit experts initially. Aside from the usual inherent challenges and obstacles, some professionals refused to participate on account of the debate surrounding DMDD’s validity as an independent diagnostic entity.^1^ Yet, it would have been extremely enlightening to gather their views, as this would certainly have nuanced certain positions. Seeing the debate still surrounding DMDD’s validity, it would benefit the scientific community to investigate this issue further.

Also, it is necessary to underscore that use of the Delphi method may result in issues being oversimplified or lacking nuance (47), as mentioned earlier. To address this issue, we added comment boxes in every section of the questionnaires. However, this generated little data, particularly in the section on pharmacological interventions. In hindsight, we might have done better to use clinical vignettes instead. Moreover, providing participants with the median score in the third round, as recommended in the Delphi Method (35), may have biased responses. Finally, as stated by Tremblay-Boudreault and Dionne (47), it is important to bear in mind that reaching a consensus does not mean that an issue has been settled once and for all. Still, the Delphi method did allow us to structure the discussion and define the remaining areas of debate (39, 52). Clearly, further research will be required to refine the results of this study. For example, future studies could examine treatment efficacy in DMDD for both psychological and pharmacological interventions.

In addition, we must keep in mind that the study was carried out over 2021 and 2022. It is possible that, in this context, major studies in the field have been published since and influence the general understanding of DMDD without this study taking account of this. Finally, the fact that this study was conducted entirely online could have hampered participation (53). Some experts might never have read our invitation to participate in the study if emails ended up among their junk mail.

Contributing to the assessment of DMDD, Mürner-Lavanchy, Kaess (54) recently published a systematic review of the psychometric instruments used. While no instrument serves as a gold standard, these authors reported several that seem useful, including the Kiddie Schedule for Affective Disorders and Schizophrenia Present and Lifetime Version, the Preschool Age Psychiatric Assessment, and the Child and Adolescent Psychiatric Assessment. However, these instruments did not meet with a consensus among our study participants, who indicated, though, that it was preferable to use clinical interviews and different validated measures.

The experts also emphasized the elements to assess, such as intensity, frequency and impact of DMDD symptoms in various contexts and relationships (e.g., parents, siblings, peers and teachers). Accordingly, it seemed appropriate to work in collaboration with the youth, the parents, and the school community. Also, participants suggest that clinicians should have access to observational reports or even to directly observe these youths in their usual environment during home-visits. Epidemiologic studies (2, 3) and clinical studies (22, 55) have found learning difficulties frequently co-occur with DMDD. Indeed, youths with severe and persistent irritability present a high rate of academic failure and learning difficulties, which justifies focusing on how they function at school (21).

Moreover, various components to be assessed regarding temper outbursts and mood (e.g., aggression, impulsivity, frustration intolerance, emotional instability). Ideally, temper outbursts should be described using a systematic approach (measuring at least the frequency, intensity, duration, triggers, environmental context, and settings). Mood, for its, part, should be observed longitudinally. In this regard, participants proposed using the Affective Reactivity Index, which has been applied in research and clinical practice to measure irritability (56). Furthermore, the families of youths who have temper outbursts tend to make accommodations to prevent these. It is important to explore caregivers’ coping strategies to DMDD symptoms, also influenced by their own emotional regulation abilities, in the course of assessment. Moreover, according to the participants, family antecedents, chronological history of mood symptoms, general functioning, and risk and protective factors, also, should be considered. To collect all of this information, it is essential for clinicians to involve in the assessment process different people who play a significant role in the life of these youths (57).

Obviously, in the course of assessment, youths should be screened more broadly for different physical and mental problems, particularly those mentioned among the DMDD exclusion criteria, namely, oppositional defiant disorder, intermittent explosive disorder, and bipolar disorder (1). According to our participants, potential contributing factors to emotion dysregulation should also be explored, including maltreatment (58), family problems (4), substance use (59), and recent negative life events (e.g., school bullying, change in custody) (60). Where differential diagnosis and comorbidities are concerned, various elements need to be taken into account: depression symptoms, suicidal behavior, sleep difficulties, anxiety symptoms, attention deficit disorder with or without hyperactivity symptoms, and comorbidity with other neurodevelopmental disorders (including specific learning disabilities). Moreover, because the idea of assessing sleep and language problems was proposed by participants in the final round of the survey, it could not be put to the rest of the group. However, it is evident that sleep difficulties (61) and language impairments (62) can have an impact on emotion regulation and the development of psychopathologies.

The section on assessment tools was the only one where no consensus was reached. Some respondents expressed a preference for clinical interviews over questionnaires. Others specified that questionnaires should be used only once potential comorbidities have been identified. In this regard, some tools, such as the Children’s Depression Rating Scale for depression symptoms (63), the Conners for ADHD symptoms (64), and the Child Behavior Checklist for emotional and behavioral problems (65) could be useful but not necessarily essential to assessing DMDD. Finally, one participant pointed out in the last round the possibility of using a questionnaire published very recently concerning DMDD specifically, namely, the Disruptive Mood Dysregulation Disorder Questionnaire (66). That said, the lack of consensus regarding evaluation tools is not a surprising finding. This issue was raised, among others, by Carlson in 1998 regarding PBD (67). She criticized the use of symptom checklists in the form of structured clinical interviews because they relied on a descriptive psychiatric paradigm that neglected contextual and developmental factors (67). According to her, a structured clinical interview could not replace longitudinal assessment with multiple informants. More recently, Galanter (68) issued the same criticisms, specifying that structured interviews “are only as good as the experience, intellect and empathic capacity of the interviewers, all of which is complicated by the perspective the interviewer has on the boundaries of bipolar disorder” (69). These critiques though related to PBD remain relevant in relation to DMDD.

Furthermore, participants proposed a four-step process for performing child psychiatric assessments: (1) interview with parents and child together; (2) meeting with parents only, preferably conducted by a different professional; (3) individual examination of child; and (4) meeting with parents and child, along with all professionals involved, to sum up the situation. This way of proceeding should be adapted to each healthcare environment.

Regarding DMDD therapy, all of the intervention targets proposed in the first round were retained, except for sensorimotor integration. The targets are presented in Table 2 by category (general concepts, emotion regulation, and interpersonal relationships). The only comment made by participants concerned the attention that should be paid to targets more specific to comorbidities. For example, if a child has comorbid DMDD and a language impairment, it is important to identify therapeutic possibilities specific to this issue.

Several experts nuanced the role of psychosocial intervention and suggested that it should apply on the results of a full-blown assessment, including developmental stage, family situation and comorbidities. Later, in comments made in the last round, several respondents expressed concerns in this regard. In the first round, some experts proposed targeted intervention techniques (e.g., chain analysis), while others recommended more comprehensive therapy programs (e.g., dialectical behavior therapy). This imbalance seemed to have bothered some participants, which might have biased part of the results. However, overall, psychoeducation, behavior-focused therapy (e.g., dialectical behavioral therapy, chain analysis, exposition, relaxation) and systemic therapy (parent management training, family therapy, parent–child interaction therapy) met consensus. Also, all proposals regarding intervention targeting parents and the family were validated by the second tour. These results echo the psychosocial interventions published in the field: interpersonal psychotherapy (70, 71), dialectical behavior therapy (72), cognitive behavioral therapy (73, 74), parent management training (74), anger management program (75), and Triple P – Positive Parenting Program (76). However, these intervention programs have their pros and cons. For example, they are more or less structured, they are more or less available in the community. Instead of adopting comprehensive intervention programs, our experts seemed to prefer using different intervention strategies depending on their patients’ needs (20), which resonates with recent proposals like those of Evans, to opt for a modular approach to treat severe irritability (77).

Other elements appeared useful to DMDD psychosocial intervention: learning emotion regulation and anger management techniques, developing coping skills. Close partnership with the school community was largely endorsed in view of generalizing learned emotional skills during therapy in children’ routine behaviors. Furthermore, our experts mentioned that the additional support of a social worker often proved useful.

One participant put forth some new ideas in the first round. He underscored the importance of establishing above all a bond of trust with the family (78). Then, he suggested addressing how to manage destructive and self-destructive behaviors with the youth in question and their family. This expert recommended using behavioral and family approaches before proposing more structured individual therapy to patients. He stressed the importance of not undertaking a structured or cognitive treatment too soon, seeing how patients were not ready emotionally to engage in the required learning (79). Also, cognitive restructuring was considered equivocal by the study participants, in addition to the duration and the format (individual/group) of intervention that should be preferred. We might imagine that these elements vary considerably across practice settings (e.g., outpatient vs. inpatient).

Regarding proposals related to pharmacological interventions, results must be interpreted with caution as this was the section with the largest number of equivocal and rejected items. First and foremost, it should be noted that only the participants who were psychiatrist were able to contribute to this part of the study. Then, the respondents themselves mentioned consensually that there was insufficient scientific evidence in this field at present to reach any decisions. They also noted that it was hard to choose the best treatment option with regard to the brevity of the assumption and the lack of context (e.g., clinical vignettes). Despite this, the experts agree on certain general guidelines. For example, it is preferable to turn to a child and adolescent psychiatrist, and if a possible a professional well-trained in pediatric mood disorders. Also, comorbidity and symptom targets can be used to guide treatment choice. Comorbidities should be treated first. For example, in the presence of comorbid ADHD, stimulants should be optimized. If DMDD is still present thereafter, other drugs may be considered as add-on. Here are the pharmacological proposals published to date regarding DMDD: stimulants (80–84), atypical antipsychotics (85, 86), mood stabilizers (84, 87), selective serotonin reuptake inhibitors (88), and atomoxetine, a selective noradrenaline reuptake inhibitor (89).

Certain drugs, such as atypical antipsychotics, mood stabilizer and anticonvulsant medications, sometimes combined (90), have been and are prescribed by proponents of PBD to reduce perceived “childhood mania” (91–94). However, since DMDD has been associated with depression, rather than bipolar disorder (13), participants appear to favor the use of SSRIs over antipsychotic. Indeed, SSRIs are a second line therapy to psychosocial interventions and medication for insomnia in youth suffering from depression, with informed consent provision about potential activation syndromes and hostility or suicidality adverse events (95). This line of reasoning follows logic and seems especially advantageous when we take into account the harmful side effects of antipsychotics on developing bodies (96, 97). Yet, the results of Findling’s study (98) are as interesting as they are worrying. The authors state that “Diagnosis of DMDD has had rapid uptake in clinical practice but is associated with increased antipsychotic and polypharmacy prescriptions and higher rates of comorbidity and inpatient hospitalization in youth with a DMDD diagnosis compared with a PBD diagnosis.” The issue of pharmacological interventions for DMDD, particularly in the context of high comorbidity, really needs to be further studied. Moreover, these results seem to indicate that even though the participants in this study emphasize the use of psychosocial treatments, currently it does not seem to be the norm in mental health practice.

### Other comments

All but one of the comments made in the first round, in the free text boxes, were validated by the participants. For example, they agreed that DMDD remained controversial and that most DMDD diagnoses did not abide by the DSM-5 rules for differential diagnosis (1). Our experts also made four proposals for future research on the subject: (i) the ICD-11 construct of ODD with emotion dysregulation should be reviewed in connection with DMDD (99, 100); (ii) childhood trauma, attachment difficulties, and parents with mental illness, including borderline personality disorder and ADHD, are associated with DMDD and their role should be explored (55, 101); (iii) complex post-traumatic stress disorder and DMDD, as well as the transition of DMDD with borderline personality disorder, should be explored (102); and (iv) the issue of the over-diagnosis of pre-pubertal bipolar disorder that led to the creation of the DMDD construct should also be reviewed in future (8, 98).

In addition, some participants had hoped that DMDD comorbidities, particularly ODD and IED, would be addressed more directly in this study. A few comments to this effect were collected in the last round. Some experts pointed out that the symptoms of these orders overlapped considerably. Other participants were critical of the fact that ODD was considered first and foremost a behavior disorder that did not sufficiently take account of mood symptoms or context. According to some participants, IED seemed extremely rare in the pediatric population. Finally, a few experts found that the differential diagnosis rules for DMDD were absurd and should be reviewed (e.g., cannot coexist with ODD, symptoms do not occur exclusively during an episode of major depressive disorder but DMDD can coexist with major depressive disorder). Future research and an APA DSM Task Force could certainly contribute to demystify these issues.

## Conclusion

In sum, the participants in our study seemed to indicate that a multidisciplinary team and an integrative approach, including family-focused interventions, should be favored to treat youths with DMDD. As much as possible, families should be offered support and professionals should work in close collaboration with the schools in communities. In this study, various psychosocial proposals are retained. A modular approach, as proposed by Evans, could be an interesting way to tackle treatment (77). The research in the field of psychosocial treatments is generally encouraging (24). Nevertheless, future studies remain necessary in this area. Furthermore, medication is, at times, a necessary option, mostly when comorbidities such as ADHD occur. However, to this date, there has been insufficient research to allow clear recommendations for DMDD in this regard. Indeed, comorbidities can widely influence DMDD treatment plans (psychosocial and pharmacological). Overall, a comprehensive clinical assessment was endorsed as the cornerstone of the therapeutic approach for youths with DMDD. We hope that this study will aid the scientific community to (i) get a better understanding of youths with DMDD symptoms, (ii) provide relevant information to clinicians, and (iii) raise new research questions and projects.

## Data availability statement

The original contributions presented in the study are included in the article/supplementary material, further inquiries can be directed to the corresponding author.

## Ethics statement

The studies involving humans were approved by the Human Research Ethics Board of Université du Québec à Montréal, in Canada (Approval no. 4953). The studies were conducted in accordance with the local legislation and institutional requirements. The participants provided their written informed consent to participate in this study. Written informed consent was obtained from the individual (s) for the publication of any potentially identifiable images or data included in this article.

## Author contributions

AB wrote the first draft of the manuscript as part of her thesis. RL reviewed all versions of the manuscript in preparation for the final draft of the article. AB and RL were the capacity of her thesis research supervisor, had full access to all the study data and ensured its integrity and the accuracy of data analysis. J-MG, J-JB, XB, and DC contributed to the interpretation of the results and to drafting the manuscript. All authors contributed to revising the manuscript and read and approved the submitted version.

## Footnotes

^1^For example, after sending a reminder about the possibility of participating in the first round, a potential participant wrote this: “I feel guilty every time I get your invitation and do not respond. It is not that I do not have time. It is that I’m not sure that I believe that DMDD is a real entity. We are struggling with how to deal with children who have problems with irritability and emotional outbursts. DMDD is neither a reliable diagnosis or an especially valid one. For that reason, I think it is premature to talk about diagnosing and treating it.”

## References

[1] American Psychiatric Association. Diagnostic and statistical manual of mental disorders DSM-5. 5th ed. Arlington: American Psychiatric Publishing (2013).

[2] Althoff RR Crehan ET He J-P Burstein M Hudziak JJ Merikangas KR. Disruptive mood dysregulation disorder at ages 13–18: results from the National Comorbidity Survey—Adolescent Supplement. J Child Adolesc Psychopharmacol. (2016) 26:107–13. doi: 10.1089/cap.2015.0038, PMID: 26771536 PMC4800387

[3] Copeland WE Angold A Costello EJ Egger H. Prevalence, comorbidity, and correlates of DSM-5 proposed disruptive mood dysregulation disorder. Am J Psychiatr. (2013) 170:173–9. doi: 10.1176/appi.ajp.2012.12010132, PMID: 23377638 PMC3573525

[4] Dougherty LR Smith VC Bufferd SJ Carlson GA Stringaris A Leibenluft E. DSM-5 disruptive mood dysregulation disorder: correlates and predictors in young children. Psychol Med. (2014) 44:2339–50. doi: 10.1017/S0033291713003115 24443797 PMC4480202

[5] Geller B Sun K Zimerman B Luby J Frazier J Williams M. Complex and rapid-cycling in bipolar children and adolescents: a preliminary study. J Affect Disord. (1995) 34:259–68. doi: 10.1016/0165-0327(95)00023-G, PMID: 8550951

[6] Wozniak J Biederman J Kiely K Ablon JS Faraone SV Mundy E. Mania-like symptoms suggestive of childhood-onset bipolar disorder in clinically referred children. J Am Acad Child Adolesc Psychiatry. (1995) 34:867–76. doi: 10.1097/00004583-199507000-00010, PMID: 7649957

[7] Moreno C Laje G Blanco C Jiang H Schmidt AB Olfson M. National trends in the outpatient diagnosis and treatment of bipolar disorder in youth. Arch Gen Psychiatry. (2007) 64:1032–9. doi: 10.1001/archpsyc.64.9.1032, PMID: 17768268

[8] Le J Feygin Y Creel L Lohr WD Jones VF Williams PG. Trends in diagnosis of bipolar and disruptive mood dysregulation disorders in children and youth. J Affect Disord. (2020) 264:242–8. doi: 10.1016/j.jad.2019.12.018, PMID: 32056757

[9] Parry PI Allison S Bastiampillai T. Reification of the paediatric bipolar hypothesis in the USA. Lancet Psychiatry. (2015) 2:14–6. doi: 10.1016/S2215-0366(14)00075-3, PMID: 26359599

[10] Hyman SE. The diagnosis of mental disorders: the problem of reification. Annu Rev Clin Psychol. (2010) 6:155–79. doi: 10.1146/annurev.clinpsy.3.022806.091532, PMID: 17716032

[11] Leibenluft E Charney DS Towbin KE Bhangoo RK Pine DS. Defining clinical phenotypes of juvenile mania. Am J Psychiatr. (2003) 160:430–7. doi: 10.1176/appi.ajp.160.3.430, PMID: 12611821

[12] Leibenluft E. Chronic irritability in children is not pediatric bipolar disorder: implications for treatment. Bipolar Disord. (2019) 22:195–6. doi: 10.1111/bdi.12881, PMID: 31820531

[13] Leibenluft E. Severe mood dysregulation, irritability, and the diagnostic boundaries of bipolar disorder in youths. Am J Psychiatr. (2011) 168:129–42. doi: 10.1176/appi.ajp.2010.10050766, PMID: 21123313 PMC3396206

[14] Masi L Guilé J-M Mille C. Dysrégulation émotionnelle et comportementale sévère chez l’enfant: évolution nosologique et affiliation aux pathologies de l’humeur. Neuropsychiatr Enfance Adolesc. (2014) 62:65–71. doi: 10.1016/j.neurenf.2014.01.013

[15] Mikita N Stringaris A. Mood dysregulation. Eur Child Adolesc Psychiatry. (2013) 22:11–6. doi: 10.1007/s00787-012-0355-9, PMID: 23229139 PMC3560944

[16] Regier DA Narrow WE Clarke DE Kraemer HC Kuramoto SJ Kuhl EA. DSM-5 field trials in the United States and Canada, part II: test-retest reliability of selected categorical diagnoses. Am J Psychiatr. (2013) 170:59–70. doi: 10.1176/appi.ajp.2012.12070999, PMID: 23111466

[17] Vidal-Ribas P Brotman MA Valdivieso I Leibenluft E Stringaris A. The status of irritability in psychiatry: a conceptual and quantitative review. J Am Acad Child Adolesc Psychiatry. (2016) 55:556–70. doi: 10.1016/j.jaac.2016.04.014, PMID: 27343883 PMC4927461

[18] Malhi GS Bell E. Fake views: DMDD, indeed! Austral New Zealand Journal of Psychiatry. (2019) 53:706–10. doi: 10.1177/0004867419863162, PMID: 31282190

[19] World Health Organization. International classification of diseases for mortality and morbidity statistics (11th revision). (2020). Available: http://www.who.int/classifications/icd/en/ (Accessed February 2020).

[20] Carlson GA Singh MK Amaya-Jackson L Benton TD Althoff RR Bellonci C. Narrative review: impairing emotional outbursts: what they are and what we should do about them. J Am Acad Child Adolesc Psychiatry. (2023) 62:135–50. doi: 10.1016/j.jaac.2022.03.014, PMID: 35358662

[21] Benarous X Iancu C Guilé J-M Consoli A Cohen D. Missing the forest for the trees? A high rate of motor and language impairments in disruptive mood dysregulation disorder in a chart review of inpatient adolescents. Eur Child Adolesc Psychiatry. (2021) 30:1579–90. doi: 10.1007/s00787-020-01636-y, PMID: 32918099

[22] Carlson GA Potegal M Margulies D Gutkovich Z Basile J. Rages—what are they and who has them? J Child Adolesc Psychopharmacol. (2009) 19:281–8. doi: 10.1089/cap.2008.0108, PMID: 19519263 PMC2856921

[23] Rao U. DSM-5: disruptive mood dysregulation disorder. Asian J Psychiatr. (2014) 11:119–23. doi: 10.1016/j.ajp.2014.03.002, PMID: 25453714 PMC4254488

[24] Hendrickson B Girma M Miller L. Review of the clinical approach to the treatment of disruptive mood dysregulation disorder. Int Rev Psychiatry. (2019) 32:202–11. doi: 10.1080/09540261.2019.1688260, PMID: 31775528

[25] Jorm AF. Using the Delphi expert consensus method in mental health research. Austral New Zealand J Psychiatry. (2015) 49:887–97. doi: 10.1177/0004867415600891 26296368

[26] Dalkey N Helmer O. Delphi technique: characteristics and sequence model to the use of experts. Manag Sci. (1963) 9:458–67. doi: 10.1287/mnsc.9.3.458

[27] Linstone HA Turoff M. The Delphi method. Reading: Addison-Wesley (1975).

[28] Lynn MR Layman EL Englebardt SP. Nursing administration research priorities: a national Delphi study. JONA. (1998) 28:7–11. doi: 10.1097/00005110-199805000-00002 9601488

[29] Avella JR. Delphi panels: research design, procedures, advantages, and challenges. Int J Dr Stud. (2016) 11:305–21. doi: 10.28945/3561

[30] Young WH Hogben D. An experimental study of the Delphi technique. Educ Res Perspect. (1978) 5:57–62.

[31] Gibson JM. Using the Delphi technique to identify the content and context of nurses' continuing professional development needs. J Clin Nurs. (1998) 7:451–9. doi: 10.1046/j.1365-2702.1998.00175.x, PMID: 9855997

[32] Procter S Hunt M. Using the Delphi survey technique to develop a professional definition of nursing for analysing nursing workload. J Adv Nurs. (1994) 19:1003–14. doi: 10.1111/j.1365-2648.1994.tb01180.x, PMID: 8056906

[33] Green B Jones M Hughes D Williams A. Applying the Delphi technique in a study of GPs’ information requirements. Health Soc Care Community. (1999) 7:198–205. doi: 10.1046/j.1365-2524.1999.00176.x, PMID: 11560634

[34] Beech B. Studying the future: a Delphi survey of how multi-disciplinary clinical staff view the likely development of two community mental health centres over the course of the next two years. J Adv Nurs. (1997) 25:331–8. doi: 10.1046/j.1365-2648.1997.1997025331.x, PMID: 9044008

[35] Keeney S McKenna H Hasson F. The Delphi technique in nursing and health research. Chichester: John Wiley & Sons (2011).

[36] Parenté FJ Anderson-Parenté JK. Delphi inquiry systems. In: Wright G Ayton P (Editords), Judgmental Forecasting. United Kingdom, Chichester: John Wiley (1987):129–56.

[37] Cavalli-Sforza V Ortolano L. Delphi forecasts of land use: transportation interactions. J Transp Eng. (1984) 110:324–39. doi: 10.1061/(ASCE)0733-947X(1984)110:3(324)

[38] Novakowski N Wellar B. Using the Delphi technique in normative planning research: methodological design considerations. Environ Plan A. (2008) 40:1485–500. doi: 10.1068/a39267

[39] Hasson F Keeney S McKenna H. Research guidelines for the Delphi survey technique. J Adv Nurs. (2000) 32:1008–15. doi: 10.1046/j.1365-2648.2000.t01-1-01567.x 11095242

[40] Sumsion T. The Delphi technique: an adaptive research tool. Br J Occup Ther. (1998) 61:153–6. doi: 10.1177/030802269806100403

[41] McKenna H Hasson F Smith M. A Delphi survey of midwives and midwifery students to identify non-midwifery duties. Midwifery. (2002) 18:314–22. doi: 10.1054/midw.2002.0327, PMID: 12473446

[42] Hill KQ Fowles J. The methodological worth of the Delphi forecasting technique. Technol Forecast Soc Chang. (1975) 7:179–92. doi: 10.1016/0040-1625(75)90057-8

[43] Eckman C. Development of an instrument to evaluate intercollegiate athletic coaches: a modified Delphi study [dissertation]. West Virginia University, Morgantown (1983).

[44] Jacobs JM. Essential assessment criteria for physical education teacher education programs: a Delphi study [dissertation]. West Virginia University, Morgantown (1996).

[45] Powell C. The Delphi technique: myths and realities. J Adv Nurs. (2003) 41:376–82. doi: 10.1046/j.1365-2648.2003.02537.x, PMID: 12581103

[46] Murry JW Jr Hammons JO. Delphi: a versatile methodology for conducting qualitative research. Rev High Educ. (1995) 18:423–36. doi: 10.1353/rhe.1995.0008

[47] Tremblay-Boudreault V Dionne C. L’approche Delphi: application dans la conception d’un outil clinique en réadaptation au travail en santé mentale. Méthodes qualitatives, quantitatives et mixtes. Montréal: Presses de l’Université du Québec (2020).

[48] Steurer J. The Delphi method: an efficient procedure to generate knowledge. Skelet Radiol. (2011) 40:959–61. doi: 10.1007/s00256-011-1145-z, PMID: 21667147

[49] Bardin L. L’analyse de contenu. 2nd ed. Paris: Presses Universitaires de France (2013).

[50] Hsieh H-F Shannon SE. Three approaches to qualitative content analysis. Qual Health Res. (2005) 15:1277–88. doi: 10.1177/1049732305276687 16204405

[51] Mayring P. Qualitative content analysis: theoretical foundation, basic procedures and software solution. Austria: Klagenfurt Press. (2014).

[52] Jones J Hunter D. Consensus methods for medical and health services research. BMJ. Br Med J. (1995) 311:376–80. doi: 10.1136/bmj.311.7001.376, PMID: 7640549 PMC2550437

[53] Cole ZD Donohoe HM Stellefson ML. Internet-based Delphi research: case based discussion. Environ Manag. (2013) 51:511–23. doi: 10.1007/s00267-012-0005-5, PMID: 23288149 PMC3581739

[54] Mürner-Lavanchy I Kaess M Koenig J. Diagnostic instruments for the assessment of disruptive mood dysregulation disorder: a systematic review of the literature. Eur Child Adolesc Psychiatry. (2023) 32:17–39. doi: 10.1007/s00787-021-01840-4, PMID: 34232390 PMC9908712

[55] Benarous X Renaud J Breton JJ Cohen D Labelle R Guilé J-M. Are youths with disruptive mood dysregulation disorder different from youths with major depressive disorder or persistent depressive disorder? J Affect Disord. (2020) 265:207–15. doi: 10.1016/j.jad.2020.01.020 32090743

[56] Stringaris A Goodman R Ferdinando S Razdan V Muhrer E Leibenluft E. The affective reactivity index: a concise irritability scale for clinical and research settings. J Child Psychol Psychiatry. (2012) 53:1109–17. doi: 10.1111/j.1469-7610.2012.02561.x, PMID: 22574736 PMC3484687

[57] De Los RA Augenstein TM Wang M Thomas SA Drabick DA Burgers DE. The validity of the multi-informant approach to assessing child and adolescent mental health. Psychol Bull. (2015) 141:858–900. doi: 10.1037/a0038498, PMID: 25915035 PMC4486608

[58] Dvir Y Ford JD Hill M Frazier JA. Childhood maltreatment, emotional dysregulation, and psychiatric comorbidities. Harv Rev Psychiatry. (2014) 22:149–61. doi: 10.1097/HRP.0000000000000014, PMID: 24704784 PMC4091823

[59] Mikolajczak M Desseilles M. Traité de régulation des émotions. Bruxelles: De Boeck Supérieur (2012).

[60] McLaughlin KA Hatzenbuehler ML. Mechanisms linking stressful life events and mental health problems in a prospective, community-based sample of adolescents. J Adolesc Health. (2009) 44:153–60. doi: 10.1016/j.jadohealth.2008.06.019, PMID: 19167664 PMC2881598

[61] Gregory AM Sadeh A. Sleep, emotional and behavioral difficulties in children and adolescents. Sleep Med Rev. (2012) 16:129–36. doi: 10.1016/j.smrv.2011.03.007 21676633

[62] Yew S O'Kearney R. Early language impairments and developmental pathways of emotional problems across childhood. Int J Lang Commun Disord. (2015) 50:358–73. doi: 10.1111/1460-6984.12142, PMID: 25556640

[63] Poznanski EO Cook SC Carroll BJ. A depression rating scale for children. Pediatrics. (1979) 64:442–50. doi: 10.1542/peds.64.4.442 492809

[64] Conners CK. Conners 3. North Tonawanda, New York: MHS (2008).

[65] Achenbach TM Rescorla LA. Manual for the ASEBA School-age Forms & Profiles. Burlington (US): University of Vermont (2001).

[66] Boudjerida A Labelle R Bergeron L Berthiaume C Guilé J-M Breton J-J. Development and initial validation of the disruptive mood dysregulation disorder questionnaire among adolescents from clinic settings. Front Psychiatr. (2022) 13:7991. doi: 10.3389/fpsyt.2022.617991, PMID: 35250652 PMC8891213

[67] Carlson GA. Mania and ADHD: comorbidity or confusion. J Affect Disord. (1998) 51:177–87. doi: 10.1016/S0165-0327(98)00179-7 10743850

[68] Galanter CA Hundt SR Goyal P Le J Fisher PW. Variability among research diagnostic interview instruments in the application of DSM-IV-TR criteria for pediatric bipolar disorder. J Am Acad Child Adolesc Psychiatry. (2012) 51:605–21. doi: 10.1016/j.jaac.2012.03.010, PMID: 22632620

[69] Parry P. 'Paediatric bipolar Disorder': why did it occur, the iatrogenic consequences, and the implications for medical ethics and psychiatric nosology. Flinders University, Australia: College of Medicine and Public Health (2021).

[70] Miller L Hlastala SA Mufson L Leibenluft E Riddle M. Interpersonal psychotherapy for adolescents with mood and behavior dysregulation: evidence-based case study. Evidence-Based Practice Child Adolescent Mental Health. (2016) 1:159–75. doi: 10.1080/23794925.2016.1247679, PMID: 29707641 PMC5915317

[71] Miller L Hlastala SA Mufson L Leibenluft E Yenokyan G Riddle M. Interpersonal psychotherapy for mood and behavior dysregulation: pilot randomized trial. Depress Anxiety. (2018) 35:574–82. doi: 10.1002/da.22761, PMID: 29719093 PMC11108175

[72] Perepletchikova F Nathanson D Axelrod SR Merrill C Walker A Grossman M. Randomized clinical trial of dialectical behavior therapy for preadolescent children with disruptive mood dysregulation disorder: feasibility and outcomes. J Am Acad Child Adolesc Psychiatry. (2017) 56:832–40. doi: 10.1016/j.jaac.2017.07.789, PMID: 28942805

[73] Kircanski K Clayton ME Leibenluft E Brotman MA. Psychosocial treatment of irritability in youth. Current Treatment Options in Psychiatry. (2018) 5:129–40. doi: 10.1007/s40501-018-0141-5, PMID: 30319935 PMC6181450

[74] Waxmonsky JG Waschbusch DA Belin P Li T Babocsai L Humphery H. A randomized clinical trial of an integrative group therapy for children with severe mood dysregulation. J Am Acad Child Adolesc Psychiatry. (2016) 55:196–207. doi: 10.1016/j.jaac.2015.12.011, PMID: 26903253 PMC4764804

[75] Dixit A Mahour P Agarwal V. Cognitive Behavioural therapy for disruptive mood dysregulation disorder. Indian Journal of Mental Health. (2020) 7:158. doi: 10.30877/IJMH.7.2.2020.158-162

[76] Byrne G Connon G. The use of standard parenting management training in addressing disruptive mood dysregulation disorder: a pilot study. J Contemp Psychother. (2021) 51:259–63. doi: 10.1007/s10879-021-09489-5

[77] Evans SC Wei MA Harmon SL Weisz JR. Modular psychotherapy outcomes for youth with different latent profiles of irritability and emotion dysregulation. Front Psych. (2021) 12:618455. doi: 10.3389/fpsyt.2021.618455, PMID: 33935825 PMC8086835

[78] Valot L Lalau J-D. L’alliance thérapeutique. Médecine des Maladies Métaboliques. (2020) 14:761–7. doi: 10.1016/j.mmm.2020.09.005

[79] Vera L. Les thérapies comportementales et cognitives chez l’enfant et l’adolescent: modèles théoriques. Paris: Elsevier Masson (2014).

[80] Waxmonsky J Pelham WE Gnagy E Cummings MR O’Connor B Majumdar A. The efficacy and tolerability of methylphenidate and behavior modification in children with attention-deficit/hyperactivity disorder and severe mood dysregulation. J Child Adolesc Psychopharmacol. (2008) 18:573–88. doi: 10.1089/cap.2008.065, PMID: 19108662 PMC2680095

[81] Winters DE Fukui S Leibenluft E Hulvershorn LA. Improvements in irritability with open-label methylphenidate treatment in youth with comorbid attention deficit/hyperactivity disorder and disruptive mood dysregulation disorder. J Child Adolesc Psychopharmacol. (2018) 28:298–305. doi: 10.1089/cap.2017.0124, PMID: 29708762 PMC6016730

[82] Baweja R Belin PJ Humphrey HH Babocsai L Pariseau ME Waschbusch DA. The effectiveness and tolerability of central nervous system stimulants in school-age children with attention-deficit/hyperactivity disorder and disruptive mood dysregulation disorder across home and school. J Child Adolesc Psychopharmacol. (2016) 26:154–63. doi: 10.1089/cap.2015.0053, PMID: 26771437 PMC4800382

[83] de la Cruz LF Simonoff E McGough JJ Halperin JM Arnold LE Stringaris A. Treatment of children with attention-deficit/hyperactivity disorder (ADHD) and irritability: results from the multimodal treatment study of children with ADHD (MTA). J Am Acad Child Adolesc Psychiatry. (2015) 54:62–70.e3. doi: 10.1016/j.jaac.2014.10.006, PMID: 25524791 PMC4284308

[84] Blader JC Pliszka SR Jensen PS Schooler NR Kafantaris V. Stimulant-responsive and stimulant-refractory aggressive behavior among children with ADHD. Pediatrics. (2010) 126:e796–806. doi: 10.1542/peds.2010-0086 20837589 PMC2956067

[85] Krieger FV Pheula GF Coelho R Zeni T Tramontina S Zeni CP. An open-label trial of risperidone in children and adolescents with severe mood dysregulation. J Child Adolesc Psychopharmacol. (2011) 21:237–43. doi: 10.1089/cap.2010.0123, PMID: 21663426

[86] Pan P-Y Fu A-T Yeh C-B. Aripiprazole/methylphenidate combination in children and adolescents with disruptive mood dysregulation disorder and attention-deficit/hyperactivity disorder: an open-label study. J Child Adolesc Psychopharmacol. (2018) 28:682–9. doi: 10.1089/cap.2018.0068, PMID: 30148656

[87] Dickstein DP Towbin KE Van Der Veen JW Rich BA Brotman MA Knopf L. Randomized double-blind placebo-controlled trial of lithium in youths with severe mood dysregulation. J Child Adolesc Psychopharmacol. (2009) 19:61–73. doi: 10.1089/cap.2008.044, PMID: 19232024 PMC2692186

[88] Towbin K Vidal-Ribas P Brotman MA Pickles A Miller KV Kaiser A. A double-blind randomized placebo-controlled trial of citalopram adjunctive to stimulant medication in youth with chronic severe irritability. J Am Acad Child Adolesc Psychiatry. (2020) 59:350–61. doi: 10.1016/j.jaac.2019.05.015, PMID: 31128268 PMC9706653

[89] Benarous X Ferrafiat V Zammit J Consoli A Gérardin P Guilé J-M. Effective use of atomoxetine to treat six inpatient youths with disruptive mood dysregulation disorder without attention deficit disorder. CNS Spectr. (2020) 25:455–7. doi: 10.1017/S1092852919001020, PMID: 31218979

[90] Dusetzina SB Weinberger M Gaynes BN Farley JF Sleath B Hansen RA. Prevalence of bipolar disorder diagnoses and psychotropic drug therapy among privately insured children and adolescents. Pharmacotherapy. (2012) 32:1085–94. doi: 10.1002/phar.1148 23208835

[91] Delbello MP Schwiers ML Rosenberg HL Strakowski SM. A double-blind, randomized, placebo-controlled study of quetiapine as adjunctive treatment for adolescent mania. J Am Acad Child Adolesc Psychiatry. (2002) 41:1216–23. doi: 10.1097/00004583-200210000-00011, PMID: 12364843

[92] Findling RL McNamara NK Gracious BL Youngstrom EA Stansbrey RJ Reed MD. Combination lithium and divalproex sodium in pediatric bipolarity. J Am Acad Child Adolesc Psychiatry. (2003) 42:895–901. doi: 10.1097/01.CHI.0000046893.27264.53, PMID: 12874490

[93] Marchand WR Wirth L Simon C. Quetiapine adjunctive and monotherapy for pediatric bipolar disorder: a retrospective chart review. J Child Adoles Psychopharmacol. (2004) 14:405–11. doi: 10.1089/cap.2004.14.405, PMID: 15650496

[94] Wagner KD Weller EB Carlson GA Sachs G Biederman J Frazier JA. An open-label trial of divalproex in children and adolescents with bipolar disorder. J Am Acad Child Adolesc Psychiatry. (2002) 41:1224–30. doi: 10.1097/00004583-200210000-00012, PMID: 12364844

[95] Hetrick SE McKenzie JE Bailey AP Sharma V Moller CI Badcock PB. New generation antidepressants for depression in children and adolescents: a network meta-analysis. Cochrane Database Syst Rev. (2021) 2021:CD013674. doi: 10.1002/14651858.CD013674.pub2, PMID: 34029378 PMC8143444

[96] Ray WA Stein CM Murray KT Fuchs DC Patrick SW Daugherty J. Association of antipsychotic treatment with risk of unexpected death among children and youths. JAMA Psychiatry. (2019) 76:162–71. doi: 10.1001/jamapsychiatry.2018.3421, PMID: 30540347 PMC6440238

[97] Libowitz MR Nurmi EL. The burden of antipsychotic-induced weight gain and metabolic syndrome in children. Front Psych. (2021) 12:623681. doi: 10.3389/fpsyt.2021.623681, PMID: 33776816 PMC7994286

[98] Findling RL Zhou X George P Chappell PB. Diagnostic trends and prescription patterns in disruptive mood dysregulation disorder and bipolar disorder. J Am Acad Child Adolesc Psychiatry. (2022) 61:434–45. doi: 10.1016/j.jaac.2021.05.016 34091008

[99] Runions K Stewart R Moore J Martinez Ladino Y Rao P Zepf F. Disruptive mood dysregulation disorder in ICD-11: a new disorder or ODD with a specifier for chronic irritability? Eur Child Adolesc Psychiatry. (2016) 25:331–2. doi: 10.1007/s00787-015-0789-y, PMID: 26578258

[100] Evans SC Burke JD Roberts MC Fite PJ Lochman JE Francisco R. Irritability in child and adolescent psychopathology: an integrative review for ICD-11. Clin Psychol Rev. (2017) 53:29–45. doi: 10.1016/j.cpr.2017.01.004, PMID: 28192774

[101] Uran P Kılıç BG. Family functioning, comorbidities, and behavioral profiles of children with ADHD and disruptive mood dysregulation disorder. J Atten Disord. (2020) 24:1285–94. doi: 10.1177/1087054715588949, PMID: 26078400

[102] de Lima Martins HA. Is disruptive mood dysregulation disorder a precursor for borderline personality disorder? Avanços em. Medicina. (2021) 1:102–3. doi: 10.52329/AvanMed.28

